# Cardiac tamponade due to coronary artery injury after left upper lobectomy

**DOI:** 10.1186/s44215-024-00143-9

**Published:** 2024-03-07

**Authors:** Toru Kawakami, Tomohiro Iwakura, Ken Chen, Jun Atsumi, Kiyomi Shimoda, Miyako Hiramatsu, Yuji Shiraishi

**Affiliations:** 1 Department of Thoracic Surgery, Fukujuji Hospital, Kiyose-shi Tokyo, Matsuyama, 204-8522 3-1-24 Japan; 2 Department of Cardiovascular Surgery, Sakakibara Heart Institute, 3-16-1 Asahi-cho Fuchu-shi, Tokyo, Japan

**Keywords:** Cardiac tamponade, Lobectomy, Lung cancer, Coronary artery injury, Staple line, Staple-related injury

## Abstract

**Background:**

Cardiac tamponade caused by coronary artery injury is an extremely rare postlobectomy complication. Herein, we present a case of cardiac tamponade due to coronary artery injury after a left upper lobectomy for lung cancer and discuss the possible cause of coronary artery injury.

**Case presentation:**

An 82-year-old man with atrial fibrillation, emphysema, chronic heart failure-associated cardiomegaly, and a history of aortic stenting for an abdominal aortic aneurysm underwent a left upper lobectomy without mediastinal nodal dissection for lung cancer. Twenty-eight hours postoperatively, he lost consciousness and went into shock vitals; computed tomography revealed cardiac tamponade. Emergency surgery was performed, which revealed a left circumflex artery laceration. Although the laceration was successfully repaired, he had a gastrointestinal perforation and developed septic shock. He died 35 days after the lung surgery. Intraoperative injury to the heart cannot be ruled out, but the site of the coronary artery injury was located far from the hilum outside the surgical field during the lobectomy. Three-dimensional computed tomography showed that the site of injury was close to the multiple firing junction of the staples that divided the anterior interlobar fissure. Two staples at the multiple firing junction, which protruded perpendicularly to the cut surface, could injure the coronary artery.

**Conclusion:**

Although we cannot rule out the possibility that the intraoperative manipulation procedures contributed to the coronary artery injury, we speculate that the protruding staples might penetrate the pericardium after lung expansion and eventually injured the coronary artery.

## Background

Cardiac tamponade caused by coronary artery injury is an extremely rare postlobectomy complication. Herein, we present a case of cardiac tamponade due to coronary artery injury after a left upper lobectomy for lung cancer and discuss the possible cause of coronary artery injury.

## Case presentation

**Fig. 1:**
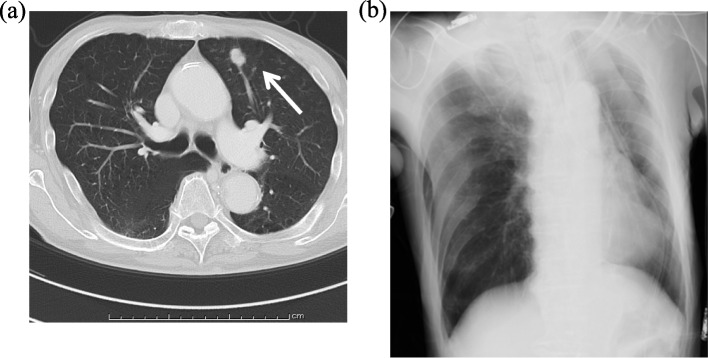
**a** Preoperative chest computed tomography. A preoperative chest computed tomography shows a 1.5-mm solid nodule with spicula in the left upper lobe (white arrow). **b** Chest radiograph taken soon after the surgery. A chest radiograph taken soon after the surgery shows that the drain tube is inserted in the direction of the pulmonary apex and that there is no worsening of cardiac enlargement

**Fig. 2:**
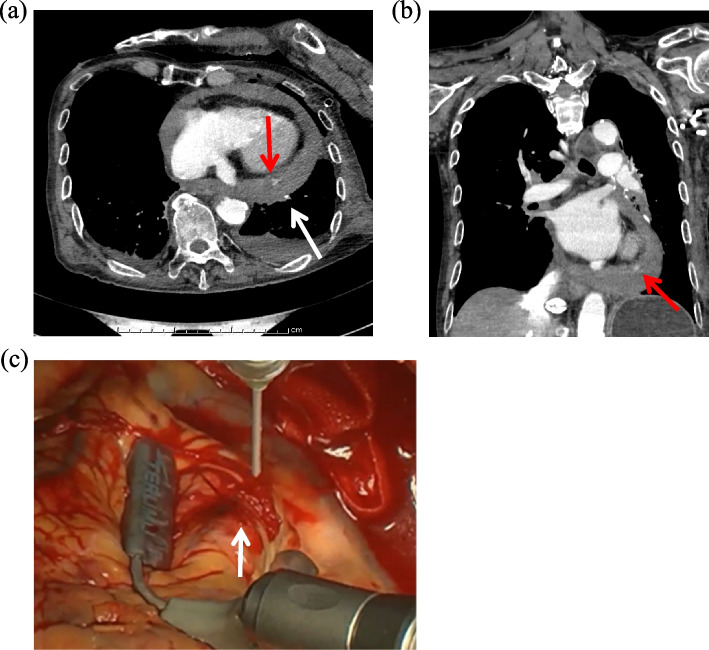
**a** Postoperative chest computed tomography in axial section. A postoperative enhanced chest computed tomography reveals hemorrhagic cardiac tamponade and extravascular leakage of contrast agent (red arrow) is shown in the pericardium. The staple line dividing the interlobar fissure is shown by the white arrow. **b** Postoperative chest computed tomography in coronal section. A postoperative enhanced chest computed tomography in coronal section reveals that the hemorrhagic tamponade and extravascular leakage of contrast agent (red arrow) is located at the apex of the heart. **c** Intraoperative image of coronary artery repair. A laceration is noted on the posterolateral branch of the left circumflex artery and blood was spurting out (white arrow)

**Fig. 3:**
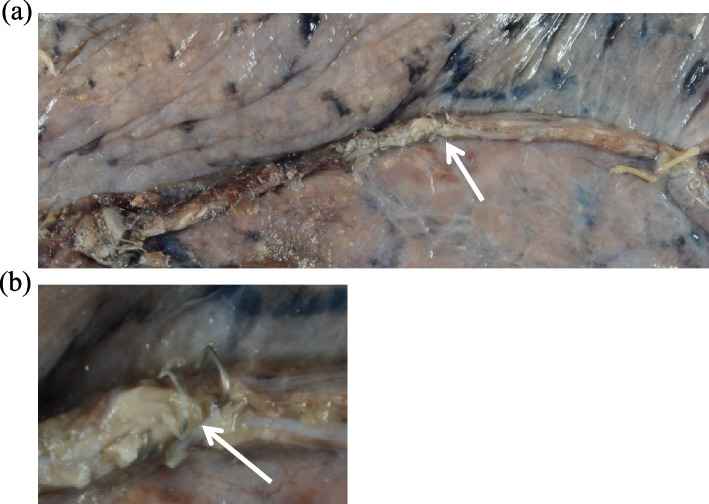
**a** Whole staple line dividing the anterior interlober fissure at the time of autopsy. The whole staple line dividing the anterior interlober fissure is shown at the time of autopsy. Two staples (white arrow) at the multiple firing junction protrude perpendicularly to the cut surface. The distal end of the staple line is ligated with silk. No malformed staples are noted even after careful palpation. **b** Enlarged image of two protruding staples at the multiple firing junction at the time of autopsy. An enlarged image of two protruding staples at the multiple firing junction are shown at the time of autopsy. One of the two staples is formed fimly to the lung and painful for us to palpate (white arrow)

An 82-year-old man with atrial fibrillation, emphysema, chronic heart failure-associated cardiomegaly, and a history of aortic stenting for an abdominal aortic aneurysm was found to have a lung tumor in the left upper lobe during a follow-up evaluation of the aortic stenting (Fig. 1a). The diagnosis was stage 1A2 (cT1bN0M0) primary lung squamous cell carcinoma. Edoxaban tosilate hydrate which had been used for the treatment of atrial fibrillation was discontinued the day before surgery. Then, continuous intravenous infusion of 10,000 units/day of heparin was started, which was terminated 9 h before surgery. No thrombus was detected on lower limb venous ultrasound, but his preoperative D-dimer was elevated to 9.3 μg/ml. He underwent a left upper lobectomy without mediastinal nodal dissection through a mini-thoracotomy. The incomplete anterior interlobar fissure was divided with three 45-mm Echelon Endopath^TM^ cartridges (Ethicon, Cincinnati, OH, USA). The operative time was 189 min, and the intraoperative blood loss was 120 mL. Chest radiograph taken soon after the surgery showed that the chest tube was inserted in the direction of the apex with suction of − 10 cmH_2_O and that there was no worsening of cardiac enlargement (Fig. 1b). A total of 10,000 units/day of heparin infusion was re-started 3 h after the surgery, and the postoperative course was uneventful for approximately 14 h until the next morning when his blood pressure decreased to approximately 85/60 mmHg. Chest drain drainage in 14 h was 100 ml and serobloody. Although his blood pressure recovered to approximately 130/80 mmHg within 2 h, 28 h after the surgery, he suddenly lost consciousness, and his blood pressure decreased to approximately 75/40 mmHg. Enhanced computed tomography (CT) of the chest revealed cardiac tamponade with extravasation of the contrast agent at the apex of the heart (Fig. 2 a and b). He was transferred to a tertiary care cardiac center where emergency surgery was performed, and a laceration was noted on the posterolateral branch of the left circumflex (LCX) artery (Fig. 2c). In addition, oozing was observed from the coronary vein and myocardia around the bleeding point of the LCX artery, and another laceration was found on the pericardium over the bleeding point of the LCX artery. No anomalies, such as an aneurysm, or significant calcifications existed around the LCX artery laceration. Although the coronary artery injury was successfully repaired, he had several postoperative complications, including gastrointestinal perforation, septic shock, respiratory failure, and renal failure. Despite intensive treatment, he died of multiple organ failure 35 days after the lung surgery. No malformed staples were identified on the staple lines even after careful palpation at the time of autopsy, but two staples at the multiple firing junction protruded perpendicularly to the cut surface (Fig. 3a). One of the two staples was formed firmly to the lung, which was painful for us to palpate (Fig. 3b).

## Discussion

Hemorrhagic cardiac tamponade is an uncommon complication after lung lobectomy. Only eight cases of hemorrhagic cardiac tamponade after lung lobectomy have been reported [1–8]. Six of the eight cases were left-side surgery, and the other two cases occurred after a right upper lobectomy (Table 1). The following causes of the bleeding site have been reported: a variant bronchial artery arising from the intrapericardial portion of the aorta or coronary artery in one case [1], a pulmonary vein retracted intrapericardially in one case [2], the ascending aorta in one case [3], the coronary artery in two cases [4, 5], and injured left ventricular wall in two cases [6, 7]. The other case involved cardiac tamponade treated with pericardiocentesis, in which the cause of bleeding was not specified [8] (Table 1).

**Table 1:**
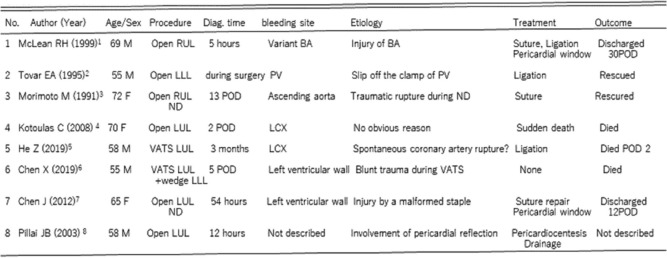
Clinical data and procedures of patients who presented with cardiac tamponade after lobectomy for lung cancer

*LCX* left circumflex

We discuss the cause of our case based on previous cases. The bleeding point in this case was not the bronchial artery, and the pulmonary vein was firmly stapled. There was no injury to the ascending aorta because no mediastinal nodal dissection was performed. Although two cases that developed bleeding from the coronary artery were from the LCX after left upper lobectomy as in our case, no obvious reason for coronary artery bleeding was identified in either case [4, 5]. The two cases of left ventricular wall injury were due to intraoperative blunt injury in one case [6] and a malformed staple in the other [7].

We speculate the cause of cardiac tamponade in this case. First is the possibility that the pericardium was damaged by the intraoperative manipulation procedures. Chen et al. previously reported a case of cardiac tamponade due to blunt trauma [6]. In our case, lacerations were found on both the pericardium and LCX artery, which led us to suspect that the cause of cardiac injury was a sharp injury. As far as we go over the video of the surgery, the intraoperative manipulation procedures including the dissection of the hilar structures and the division of the interlobar fissure were performed around the hilum. In addition, vessel staplings were performed after vessels were fully dissected, and traction during the surgery was kept minimal. This may be due to the fact that the operation was performed under direct view through the fourth intercosal lateral mini-thoracotomy. On the other hand, the site of bleeding was the apex of the heart and far from the site of intraoperative manipulation. Nevertheless, we cannot rule out the possibility that the pericardium was injured outside of the area where the scope filmed; however, we think it unlikely that any technical errors could occur intraoperatively.

**Fig. 4:**
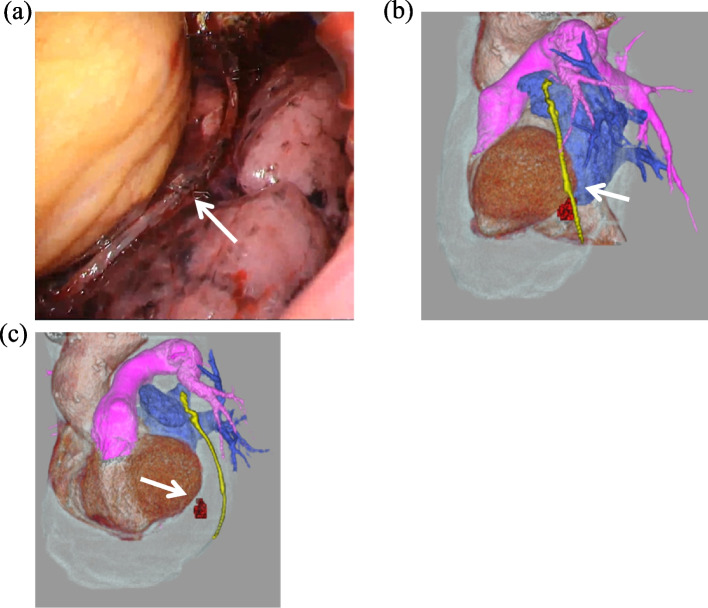
**a** Intraoperative image of the staple-line that divided the anterior interlober fissure. An intraoperative image of the staple-line that divide the anterior interlober fissure is shown. Although some staples (white arrow) protrude perpendicularly to the cut surface, no malformed staples are noted. **b** Constructed three-dimensional computed tomography. A three-dimensional computed tomography image is constructed using postoperative enhanced computed tomography. The contrast extravasation (shown in red) is on the staple line dividing the interlobar fissure (shown in yellow). The multiple firing junction is indicated by the white arrow. **c** Side image of constructed three-dimensional computed tomography. A side image of the constructed three-dimensional computed tomography is shown. This image reveals that the top edge of contrast extravasation is the bleeding point (white arrow), which is close to the multiple firing junction

Second, Chen J et al. [7] reported a unique case of left ventricular wall injury due to an “L-shaped” malformed staple on the staple line. In our case, although two staples at the multiple firing junction protruded perpendicularly to the cut surface, no malformed staples were identified on the staple lines on the video of surgery (Fig. 4a) and at the time of autopsy. A postoperative enhanced CT revealed that the bleeding point was close to the staple line. Then, we looked at the positional relationship between the staple line and the bleeding site based on a constructed three-dimensional (3D) image of the postoperative CT (Fig. 4 b and c). The site of the coronary artery injury was near the staple line that divided the anterior interlobar fissure on 3D-CT. Additionally, the site of the coronary artery injury was close to the multiple firing junction of staples dividing the anterior interlobar fissure.

Yamashita et al. [9] reported a case of cardiac tamponade after a wedge resection of the left lung due to confluence of the pulmonary stapling line. In addition, Yamano et al. [10] reported a case of bleeding from the chest wall due to a sharp protruding edge of the endostapler after a wedge resection. These reports confirmed that protruding staples can cause tissue damage after lung expansion. In our case, the autopsy revealed protruding staples at the multiple firing junction dividing the anterior interlobar fissure (Fig. 3a and 3b). In addition, the intraoperative image of the staple lines dividing the anterior interlobar fissure revealed that two staples protruded perpendicularly to the cut surface without obvious malformed staples (Fig. 4a). We, therefore, speculate that these protruding staples penetrated the pericardium after lung expansion, and eventually injured the coronary artery. In previous reports, this mechanism was observed after a wedge resection, but our case raised the possibility that it could also occur in patients after lobectomy with a larger free space. His emphysema and cardiomegaly may have contributed to this fatal complication.

We cannot rule out the possibility that the intraoperative manipulation procedures contributed to the coronary artery injury. However, we think it more likely that the protruding staples might penetrate the pericardium after lung expansion and eventually injured the coronary artery. We suggest that it might be more preferrable to use a single stapler cartridge for dividing the anterior interlobar fissure of the left lung. Should more than one stapler be necessary, we advocate that the multiple firing junction be covered with pericardial fat or TachoSil® (CSL Behring, Tokyo, Japan), as Yamashita and colleagues [9] have advocated.

## Data Availability

Not applicable.

## Abbreviations

CT: Computed tomography
LCX: Left circumflex
3D: Three-dimensional
